# Immediate changes during dysesthesia-matched transcutaneous electrical nerve stimulation in refractory neuropathic pain: a retrospective observational case series

**DOI:** 10.1186/s12984-026-02084-6

**Published:** 2026-07-03

**Authors:** Kentaro Kawamura, Seiji Etoh, Makoto Ueno, Rintaro Ohama, Takashi Hoei, Shintaro Hagihara, Kei Enohata, Megumi Shimodozono

**Affiliations:** 1 Department of Rehabilitation and Physical Medicine, Graduate School of Medical and Dental Sciences, Kagoshima University, 8-35-1, Sakuragaoka, Kagoshima, 890-8520 Japan; 2 Division of Rehabilitation, Kagoshima University Hospital, 8-35-1, Sakuragaoka, Kagoshima, 890-8520 Japan; 3 Department of Anesthesiology and Pain Medicine, Kagoshima University Hospital, 8-35-1, Sakuragaoka, Kagoshima, 890-8520 Japan

**Keywords:** Neuropathic pain, Dysesthesia, Transcutaneous electrical nerve stimulation, Neuromodulation, Trigeminal neuropathy, Rehabilitation

## Abstract

**Background:**

Dysesthesia—such as tingling and numbness—remains refractory to pharmacological treatment and poses therapeutic challenges within neuropathic pain. Dysesthesia-matched transcutaneous electrical nerve stimulation (DM-TENS) is a perceptually guided neuromodulation approach in which stimulation parameters are iteratively adjusted to synchronize with the perceived temporal patterns and intensity of the patient’s abnormal sensory experience. However, immediate responses to DM-TENS across heterogeneous neuropathic pain conditions, as well as the feasibility of both direct and indirect (remote-site) stimulation approaches have not been described.

**Methods:**

This retrospective observational case series included consecutive patients with refractory neuropathic pain referred from the pain clinic to the rehabilitation department at our university hospital between December 2024 and August 2025. Dysesthesia intensity was assessed before and during stimulation using the Numerical Rating Scale (NRS), and pain quality was evaluated using the Short-Form McGill Pain Questionnaire-2 (SF-MPQ-2) as an exploratory measure. Within-session changes were examined using session-level linear mixed-effects models, with aggregated non-parametric analyses performed as sensitivity analyses. As an exploratory analysis, within-patient consistency of stimulation parameters (frequency, pulse width, and intensity) across repeated sessions was evaluated using intraclass correlation coefficients (ICCs).

**Results:**

DM-TENS was well tolerated in all participants, with no stimulation-related adverse events requiring session termination. Fifteen patients underwent 66 DM-TENS sessions, of which 64 provided NRS data. A session-level linear mixed-effects model showed significantly lower NRS scores during stimulation (*p* < 1 × 10⁻^8^). Aggregated sensitivity analyses showed a median NRS change of − 2.0 (interquartile range − 2.75 to − 1.43). Indirect stimulation was applied only in trigeminal neuropathic presentations; lower within-session symptom scores were observed during palm stimulation, and these findings should be considered exploratory. SF-MPQ-2 total scores were also significantly lower during stimulation (*p* = 0.002). Stimulation parameters showed good to excellent within-patient consistency across repeated sessions (ICC range 0.78–0.85).

**Conclusions:**

In this retrospective observational case series, lower within-session symptom scores were observed during DM-TENS across heterogeneous neuropathic pain conditions, including trigeminal, spinal, and peripheral presentations. These findings raise the hypothesis that individualized, perceptually guided adjustment of stimulation parameters warrants prospective evaluation.

**Supplementary Information:**

The online version contains supplementary material available at https://doi.org/10.1186/s12984-026-02084-6.

## Introduction

Neuropathic pain can result from a wide variety of peripheral and central nervous system disorders, including spinal cord injury, radiculopathies, medication-induced neuropathies, and trigeminal neuralgia, leading to chronic dysesthetic symptoms such as painful tingling, numbness, and prickling sensations [1]. Although pharmacological treatments including anticonvulsants, antidepressants, and opioids are commonly employed as first-line therapies, their efficacy, tolerability, and long-term safety remain suboptimal in many patients [2, 3]. Consequently, there is increasing interest in non-pharmacological strategies for the management of neuropathic pain [4–6]. Transcutaneous electrical nerve stimulation (TENS) is a safe non-pharmacological modality for neuropathic pain and has demonstrated moderate-certainty evidence of reducing pain intensity without serious adverse events in systematic reviews [7, 8]. However, its therapeutic effectiveness remains limited for refractory symptoms frequently associated with neuropathic pain, such as tingling and numbness [9].

To overcome this limitation, a novel and individualized approach called Dysesthesia-Matched TENS (DM-TENS) has been developed [10–12]. This approach customizes stimulation parameters based on the individual characteristics of dysesthesia. Compared to conventional high-frequency TENS, DM-TENS has been reported to improve allodynia, tingling, and mechanical hypoesthesia, suggesting its potential relevance for refractory dysesthesia, primarily in patients with spinal disorders [10].

However, immediate within-session responses to DM-TENS across heterogeneous neuropathic pain conditions remain insufficiently characterized. During routine clinical practice, we observed that trigeminal symptoms could change even when stimulation was delivered away from the symptomatic region. This observation motivated the exploratory assessment of indirect stimulation applied to remote sites in patients with trigeminal neuropathic symptoms.

The present study aimed to describe within-session changes during DM-TENS across heterogeneous neuropathic pain conditions. We further explored the feasibility of both direct and indirect stimulation approaches.

## Methods

### Study design and patients

This retrospective observational case series included consecutive patients with dysesthesia who were referred from the Department of Anesthesiology (Pain Clinic) to the Department of Rehabilitation at Kagoshima University Hospital between December 2024 and August 2025. Although most cases involved chronic or treatment-refractory neuropathic pain, one acute case of trigeminal-territory dysesthesia occurring in the early-phase of Bell’s palsy was also included, reflecting real-world consecutive referrals. Detailed patient characteristics are shown in Supplementary Table S1 (Additional File 1). This study was conducted in accordance with the Declaration of Helsinki and involved DM-TENS delivered as part of routine clinical care. It was approved by the Ethics Committee of Kagoshima University Hospital (Approval No. 240306).

The requirement for informed consent was waived due to the retrospective nature of the study. Information regarding the study was disclosed via institutional bulletin boards and the hospital website, and an opt-out consent process was employed. Patients were given the opportunity to decline participation. All data were anonymized to ensure confidentiality. This study was reported in accordance with the STROBE (Strengthening the Reporting of Observational Studies in Epidemiology) guidelines for observational studies.

### Outcome measures

This study evaluated changes in dysesthesia and neuropathic pain associated with DM-TENS across different etiologies and stimulation configurations. Dysesthesia intensity was assessed using either an 11-point Numerical Rating Scale (NRS) or a 100-mm Visual Analogue Scale (VAS). Pain quality was assessed using the Short-Form McGill Pain Questionnaire-2 (SF-MPQ-2) [13], which comprises four pain descriptor subscales—continuous, intermittent, neuropathic, and affective—as well as a total score, allowing multidimensional assessment of pain quality beyond intensity. Although originally designed to assess symptoms over the preceding week, the SF-MPQ-2 was administered immediately before DM-TENS to capture current qualitative characteristics of pain at the time of stimulation. These assessments were performed immediately before (“Pre”) and during (“During”) DM-TENS in a session. This exploratory use of the SF-MPQ-2 was adopted because no validated instrument specifically designed to assess ultra-short–term changes in pain quality was available in routine clinical practice at the time of data collection. The “During” assessment was performed after the initiation of TENS, at a time point when symptom changes had clinically stabilized during ongoing stimulation.

Neuropathic pain was classified at the patient level based on the presumed anatomical origin of symptoms, taking into account clinical diagnosis, pain distribution, and surgical or medical history. Three categories were defined: trigeminal neuropathic pain, spinal neuropathic pain, and peripheral neuropathic pain. In patients with spinal disorders, radiculopathy and myelopathy were not always clearly distinguishable, particularly in postoperative or chronic compressive conditions; therefore, overlapping presentations were categorized under spinal neuropathic pain. Because some patients experienced neuropathic pain affecting multiple anatomical regions, they could be assigned to more than one neuropathic pain category.

Pain locations were categorized into three anatomical regions: face, upper limb, and lower limb. Stimulation condition was classified as “direct”, defined as stimulation delivered over the painful region, or “indirect”, defined as stimulation delivered to a remote, non-painful site. Indirect stimulation was used only for facial symptoms, typically applied to the palm, with lower-limb stimulation used only in one initial case. Symptoms in the upper and lower limbs were treated only with direct stimulation. No patient received both direct and indirect stimulation for the same anatomical pain region.

In this observational case series, the primary outcome was the change in NRS. Changes in SF-MPQ-2 were evaluated as an exploratory outcome to assess pain quality, and all other secondary analyses were considered exploratory. To assess the feasibility and safety of DM-TENS, session completion, acceptability of the therapy, and any adverse effects related to the intervention were recorded.

### DM-TENS intervention

#### Stimulation device and electrodes

DM-TENS was delivered using a commercially available stimulator (ESPURGE; Ito Co., Ltd., Tokyo, Japan) that provides adjustable biphasic pulses. Stimulation was applied through self-adhesive gel electrodes (Ito Gelpad, sizes S (φ32 mm)/M (50 × 50 mm)/L (50 × 90 mm), Ito Co., Ltd., Tokyo, Japan). For limb symptoms, electrodes were positioned on opposite sides to “sandwich” the most affected area, with the distance between electrode centers kept within approximately 10–20 cm, even when the symptomatic area was extensive. The size of the gel pads was selected according to the spatial extent of the symptomatic area. For indirect stimulation applied to the palm for facial symptoms, two medium-sized gel pads were placed on the palmar surface—one near the wrist and the other near the base of the fingers—with a small gap between them.

#### Adjustment procedure of stimulation parameters

Stimulation parameters were individually adjusted based on real-time verbal feedback from each patient. The aim was to identify stimulation settings associated with maximal alleviation of dysesthesia or pain. Frequency was adjusted before pulse width in all sessions. Detailed parameter-adjustment procedures are provided in Supplementary Fig. S1 (Additional File 2).

##### Direct application to symptomatic regions

For direct DM-TENS, electrodes were placed over the symptomatic region. Stimulation was initiated at a frequency of 100 Hz and a pulse width of 50 µs, and current intensity was gradually increased until the patient clearly perceived the electrical sensation. Frequency was then adjusted to match the perceived rhythm or qualitative features of the dysesthesia. Once the dysesthesia diminished or disappeared, the current was progressively reduced to a level slightly below the sensory threshold. If dysesthesia remained, the frequency was changed at subthreshold intensity to identify the optimal setting for symptom relief. When further residual symptoms remained, the pulse width was increased in 10-µs steps to further optimize symptom alleviation.

##### Indirect application for facial symptoms

For facial dysesthesia and pain within the trigeminal nerve distribution, electrodes were placed on the palm of the ipsilateral or contralateral hand. As in direct DM-TENS, stimulation started at 100 Hz and 50 µs, and stimulation intensity was increased until a clear sensation was perceived in the palm. The stimulation frequency was then adjusted to identify settings associated with a perceived sense of relief or a spreading/soothing sensation in the facial region. The pulse width was then incrementally adjusted to further optimize symptom alleviation. Once maximal symptom alleviation was achieved, the stimulation intensity was gradually reduced to the lowest level at which this alleviation was maintained. In this indirect approach, stimulation intensity was adjusted within tolerable limits and could be maintained above the sensory threshold when this was associated with greater facial symptom relief.

##### Stabilization and “during” assessment

Once the optimal stimulation parameters were identified, stimulation was continued under the same settings without further adjustment. In patients with multiple sessions, stimulation parameters were re-optimized at each session rather than fixed across sessions. During each session, patients were repeatedly asked whether symptoms were still improving or had reached a plateau. The “during” assessment was performed when the patient reported no further symptom change, which was considered clinically stabilized. No fixed minimum stimulation duration was predefined, as the time required to reach stabilization varied across individuals and symptom profiles. All sessions were conducted by one of two clinicians following the same standardized adjustment protocol. In practice, the median stimulation duration was 20 min overall (IQR 15–30 min).

### Data preprocessing and dataset construction

Pain outcome measures were harmonized prior to analysis to ensure comparability across assessments. When VAS was used, values were converted to NRS-equivalent scores by dividing by 10, without rounding, thereby minimizing information loss associated with scale conversion.

Linear rescaling of continuous pain outcomes is an accepted harmonization method to enable pooled statistical analyses when original scale ranges differ, as supported by empirical validation studies and real-world registry applications [14, 15].

When dysesthesia was rated separately on the left and right sides, the mean of the two values was used for the primary analysis; when only unilateral ratings were available, the single value was retained.

Although both NRS and SF-MPQ-2 assessments were planned for each session, not all sessions included both measures due to clinical time constraints or patient burden. Only sessions with complete pre–during pairs were included for each respective outcome, and no missing data were imputed; sessions lacking either a pre- or during value were excluded.

As a result, NRS ratings were available for 64 sessions from 15 patients, and SF-MPQ-2 assessments were available for 40 sessions from 11 patients, including 38 sessions with concurrent NRS ratings and two SF-MPQ-2–only indirect facial sessions.

Of the 64 sessions included in NRS-based analyses, 30 sessions (46.9%) used original NRS ratings, whereas 34 sessions (53.1%) used VAS values converted to NRS-equivalent scores. In direct stimulation sessions (n = 40), 15 sessions (37.5%) used original NRS ratings and 25 sessions (62.5%) used converted VAS values. In indirect stimulation sessions (n = 24), 15 sessions (62.5%) used original NRS ratings and 9 sessions (37.5%) used converted VAS values.

#### Definition of within-session change (Δ)

For both NRS and SF-MPQ-2 outcomes, within-session change (Δ) was defined as the during-stimulation score minus the pre-stimulation score (During − Pre), with negative values indicating symptom reduction.

#### Aggregation and handling of repeated sessions

Given that participants contributed different numbers of repeated DM-TENS sessions, two complementary analytical approaches were adopted. The primary inferential analyses were conducted at the session level using linear mixed-effects models, which explicitly accounted for within-subject correlation by including subject as a random intercept.

In addition, aggregated analyses were performed as sensitivity analyses to examine the robustness of the findings under a more conservative data structure. Specifically, for each subject × pain region × stimulation condition (direct stimulation vs indirect stimulation), pre-stimulation values of NRS and SF-MPQ-2 were summarized using the median across all available sessions, and the same procedure was applied to during-stimulation values to derive a single representative paired pre–during observation per analytical unit.

These aggregated datasets were used primarily for non-parametric analyses of within-session change and between-condition comparisons. The purpose of aggregation was not to replace the session-level mixed-effects models, but to confirm that the observed changes were not driven disproportionately by individuals with a higher number of stimulation sessions.

For patients with facial neuropathic pain treated with indirect DM-TENS, aggregation was performed at the subject level irrespective of the exact remote stimulation site (e.g., palm or leg), because all sessions targeted the same symptomatic facial region.

A single participant could also contribute multiple aggregated units if different anatomical regions were clinically affected.

As indirect stimulation was used only for facial symptoms, and limb symptoms were treated only with direct stimulation, stimulation condition and pain region were not crossed and could not be evaluated as independent factors.

### Statistical analysis

#### Primary outcome analyses

##### Session-level linear mixed-effects models

Linear mixed-effects models were fitted separately for NRS and SF-MPQ-2 outcomes using unaggregated session-level measurements. Fixed effects included session (Pre vs During), stimulation condition (Direct vs Indirect), and their interaction, with subject specified as a random intercept. Pain region was not included as a fixed effect because it was completely confounded with stimulation condition and therefore not identifiable within the same model. Fixed effects in the mixed-effects models were evaluated using Type III analysis of variance with Satterthwaite’s approximation for degrees of freedom. For mixed-effects models, effect sizes (r) were derived from F statistics and denominator degrees of freedom using the transformation r = √(F/(F + df)). Statistical significance was defined as *p* < 0.05.

##### Aggregated non-parametric analyses

Continuous outcomes are presented as median (interquartile range). Given that the outcome measures are ordinal in nature, non-parametric paired tests were used for within-unit comparisons. The primary within-session change in symptoms was evaluated using Wilcoxon signed-rank tests on aggregated paired Pre- and During-stimulation values. To facilitate clinical interpretation, the corresponding change scores (Δ = During − Pre) were additionally summarized descriptively (median, IQR), but Δ values were not used as the formal statistical test input for the primary within-unit analysis.

#### Comparison of change between direct and indirect stimulation

To examine whether stimulation condition (Direct vs. Indirect) was associated with different magnitudes of change, aggregated Δ values for each outcome were compared between conditions using Mann–Whitney U tests (Wilcoxon rank-sum test) as an exploratory analysis. Because stimulation condition and pain region were not independent, between-condition comparisons should be interpreted cautiously, as they do not isolate a pure stimulation-site contribution. Effect size r was derived from the standardized Wilcoxon and Mann–Whitney test statistics. *p*-values for SF-MPQ-2 subscale analyses were adjusted for multiple comparisons using the Holm–Bonferroni method.

#### Analyses of stimulation parameters across pain regions

For analyses of stimulation parameters, all available DM-TENS sessions with complete parameter records were included. This yielded 66 sessions from 15 participants, comprising 26 facial, 20 upper limb, and 20 lower limb sessions. Differences in stimulation parameters across pain regions were primarily assessed using linear mixed-effects models at the session level, with subject specified as a random intercept to account for repeated sessions within individuals. As a sensitivity analysis, subject-level median values were compared across regions using Kruskal–Wallis tests followed by Bonferroni-adjusted pairwise Mann–Whitney U tests.

#### Associations between stimulation parameters and symptom measures

Associations between baseline scores (pre-stimulation NRS and SF-MPQ-2 total score) or within-session change scores (ΔNRS and ΔSF-MPQ-2 total score) and stimulation parameters (frequency, pulse width, and intensity) were examined using Spearman’s rank correlation coefficients.

#### Analyses by neuropathic pain type

To explore whether the magnitude of within-session dysesthesia reduction differed according to neuropathic pain type, changes in dysesthesia intensity were quantified as ΔNRS (During − Pre) at the session level. Linear mixed-effects models with subject specified as a random intercept were fitted to account for repeated measurements within individuals, with neuropathic pain type (trigeminal, spinal, peripheral) included as a fixed effect. As sensitivity analyses, ΔNRS values were additionally aggregated at the subject-by-pain-type level using the median, and group differences were assessed using the Kruskal–Wallis test. Furthermore, linear mixed-effects models including stimulation condition and/or anatomical pain region as additional fixed effects were fitted to evaluate the robustness of the findings. These models were interpreted cautiously, given the strong coupling between pain type, anatomical region, and stimulation configuration in this case series.

#### Consistency of TENS parameter settings (ICC analysis)

Because DM-TENS is often applied repeatedly in clinical practice, the consistency of stimulation parameter settings across repeated sessions within the same subject and pain region was evaluated. Analyses were restricted to subject–pain region combinations with at least two DM-TENS sessions, yielding 62 sessions from 11 patients and forming 15 subject–region clusters. Consistency was quantified using one-way random-effects intraclass correlation coefficients (ICC(1,1)) [16]. ICCs were estimated from random-intercept models fitted by restricted maximum likelihood and computed as the ratio of between-cluster variance to total variance (between + within). Ninety-five percent confidence intervals were obtained using parametric bootstrap resampling (2000 iterations).

#### Responder analyses

For the 64 sessions in which NRS was measured, as well as for the 19 aggregated units, two outcome metrics were examined: the absolute change in NRS (ΔNRS) and the percentage reduction from baseline, calculated as (Pre − During)/Pre × 100. Responders were defined according to IMMPACT benchmarks, with a ≥ 30% reduction in NRS indicating a moderately important improvement and a ≥ 50% reduction indicating a substantial improvement [17]. In addition, an absolute reduction of ≥ 2 points was examined as a sensitivity definition of clinically important improvement, based on an anchor-based threshold of clinically important change [18]. Responder analyses were conducted as exploratory analyses to facilitate clinical interpretation of immediate within-session changes in NRS.

### Software

Statistical analyses were performed using EZR (version 1.70; Saitama Medical Center, Jichi Medical University, Saitama, Japan) and R (version 4.5.2; R Foundation for Statistical Computing, Vienna, Austria). Procedures requiring advanced functionality, including Type III ANOVA, effect-size estimation, and intraclass correlation coefficient analyses, were performed in R using lme4, lmerTest, and effectsize packages.

### Use of AI assistance

An AI-assisted language model (ChatGPT, OpenAI; accessed 2025–2026) was used to support drafting of R code templates and refinement of English phrasing. All analytical decisions, final code, and manuscript content were determined and verified by the authors.

## Results

### Patient and session characteristics

A total of 15 patients with neuropathic pain were included.

Regarding feasibility and safety, no participants discontinued DM-TENS sessions due to discomfort or intolerance during stimulation. No skin-related adverse effects associated with electrode placement or electrical stimulation were observed. However, in one patient with spinal cord–related neuropathic pain, a transient exacerbation of symptoms was observed for a short period after the session.

Patient and session characteristics are summarized in Table 1. Detailed patient characteristics and stimulation-related clinical observations are shown in Supplementary Table S1 (Additional File 1).

**Table 1:** Patient characteristics, session summary, and stimulation parameters

Section	Variable	Value
Patient-level characteristics		
Demographics	Number of patients	15
	Age, years	62 (range 13–78)
	Sex, n (male / female)	6/9
Neuropathic pain classification†	Trigeminal neuropathic pain, n	7
	Spinal neuropathic pain, n	6
	Peripheral neuropathic pain (non-spinal), n	6
Session-level characteristics		
Session summary	Total number of sessions	66
	Sessions per patient	4 [2–7] (range 1–9)
Pain region	Face, n (%)	26 (39.4%)
	Upper limb (arm/hand), n (%)	20 (30.3%)
	Lower limb (leg/foot), n (%)	20 (30.3%)
Stimulation condition	Direct (D), n (%)	40 (60.6%)
	Indirect (ID), n (%)	26 (39.4%)
Stimulation parameters	Frequency, Hz	150 [50–200]
	Pulse width, μs	60 [50–70]
	Intensity, mA	22 [15–32]
Evaluable datasets	NRS pre–during pairs, sessions	64
	SF-MPQ-2 pre–during pairs, sessions	40
	Sessions with both outcomes available	38‡

^†^ Some patients presented with more than one type of neuropathic pain (e.g., trigeminal and spinal, or multiple peripheral sites); therefore, neuropathic pain categories are not mutually exclusive. ‡ Two SF-MPQ-2 sessions were conducted on days without concurrent NRS assessments. n refers to the number of patients for patient-level variables and the number of sessions for session-level variables

Detailed information regarding disease diagnosis and symptom duration of each patient is provided in Supplementary Table S1 (Additional File 1)

*NRS*, numerical rating scale; *SF-MPQ-2*, Short-Form McGill Pain Questionnaire–2. *Data presentation:* Continuous variables are presented as median [Q1–Q3] unless otherwise stated

Overall, 66 DM-TENS sessions were available for descriptive analyses and stimulation-parameter analyses. Of these, 63 sessions (95.5%) were conducted in the outpatient setting, whereas three sessions (4.5%) were indirect facial (trigeminal) sessions performed in a single patient during an orthopedic inpatient admission for rotator cuff tear. Pain regions were categorized as face (26 sessions, 39.4%), upper limb (arm/hand; 20 sessions, 30.3%), and lower limb (leg/foot; 20 sessions, 30.3%). Stimulation was delivered either directly to the painful region (Direct; 40 sessions, 60.6%) or indirectly to a remote site (Indirect; 26 sessions, 39.4%).

For analyses of dysesthesia intensity, 64 sessions provided complete pre–during paired NRS assessments, and were therefore included in NRS-based figures and models; these comprised 40 Direct and 24 Indirect sessions, yielding 19 aggregated subject–region–condition units (12 Direct, 7 Indirect) for non-parametric analyses.

For pain quality outcomes, 40 sessions provided complete pre–during paired SF-MPQ-2 assessments, yielding 13 aggregated subject-region-condition units (8 Direct, 5 Indirect) for non-parametric analyses. Of these, 38 sessions were obtained concurrently with NRS evaluations; two additional SF-MPQ-2–only sessions involved facial symptoms treated with indirect stimulation and were therefore included only in SF-MPQ-2-specific and stimulation-parameter analyses. A schematic overview of the analyzable datasets and aggregation structure is provided in Supplementary Fig. S2 (Additional File 2).

### Changes in dysesthesia intensity (NRS) during DM-TENS

**Fig. 1:**
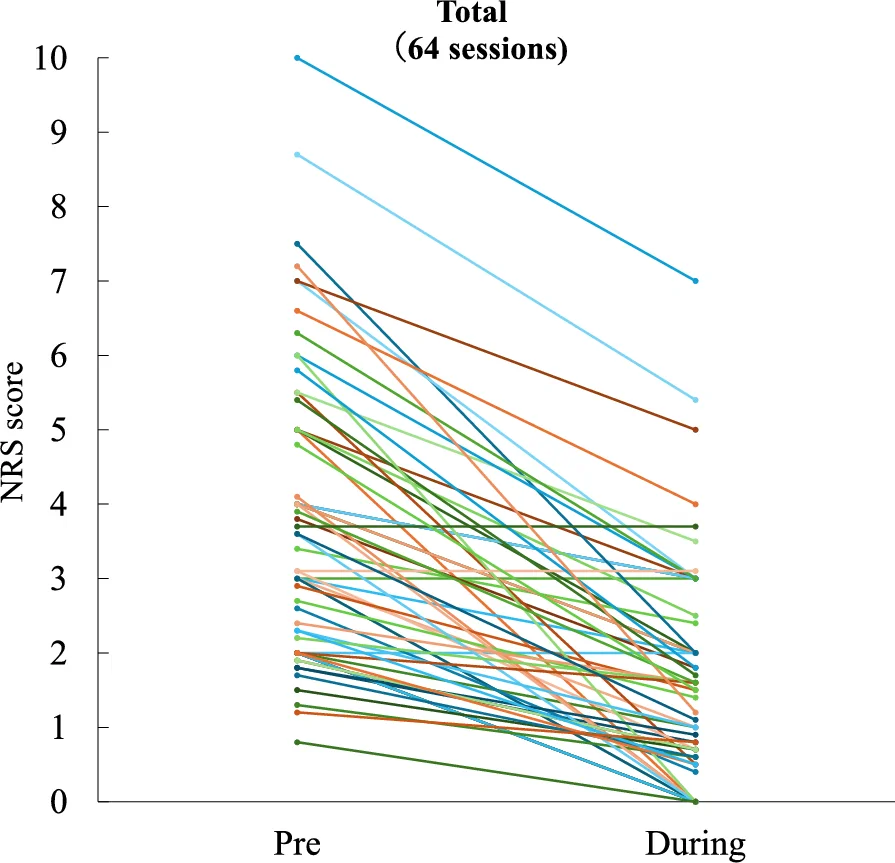
Session-level changes in dysesthesia intensity assessed by the Numerical Rating Scale (NRS) before (Pre) and during (During) dysesthesia-matched transcutaneous electrical nerve stimulation (DM-TENS). Paired lines represent individual stimulation sessions (total: 64 sessions). Only sessions with complete paired pre–during NRS data are shown; two additional sessions contributed SF-MPQ-2 and stimulation-parameter data but lacked NRS data and were therefore not included here

**Fig. 2:**
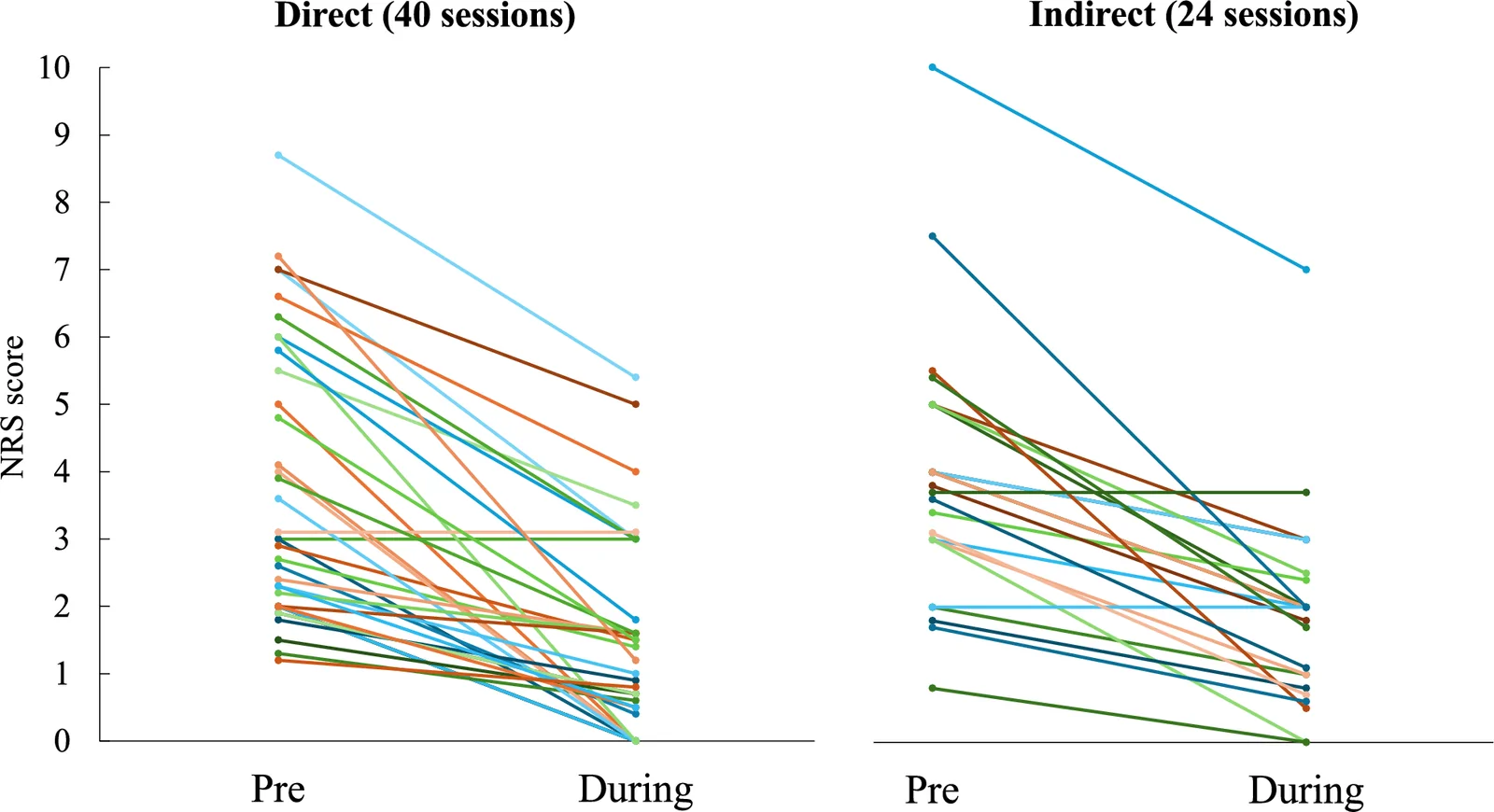
Session-level changes in dysesthesia assessed by the NRS before (Pre) and during (During) DM-TENS, shown separately for the direct (left) and indirect (right) stimulation conditions. Panels separate direct stimulation applied to the symptomatic region (left; 40 sessions) and indirect stimulation delivered to a non-symptomatic remote site (right; 24 sessions). Indirect stimulation was applied predominantly to the palm and occasionally to the lower leg in patients with facial symptoms. Indirect stimulation counts in this figure are restricted to sessions with complete paired NRS data; two additional indirect facial sessions contributed SF-MPQ-2 and stimulation-parameter data but lacked NRS and were therefore not included here. Because stimulation condition (direct vs indirect) was completely confounded with pain region, these panels should not be interpreted as representing independent comparative groups

Session-level changes in dysesthesia intensity across all 64 DM-TENS sessions are shown in Fig. 1. NRS scores decreased from Pre to During across both Direct and Indirect stimulation conditions. Session-level NRS changes stratified by stimulation condition (Direct vs Indirect) are shown in Fig. 2.

### Session-level mixed-effects model

A linear mixed-effects model including all available session-level NRS observations indicated a statistically significant main effect of session (Pre vs During), with lower NRS scores during stimulation compared with pre-stimulation (estimate − 2.28, SE 0.34, t =  − 6.68, *p* < 1 × 10⁻^8^). Neither the main effect of stimulation condition (*p* = 0.51) nor the session × condition interaction (*p* = 0.65) reached statistical significance.

### Aggregated non-parametric sensitivity analyses

**Fig. 3:**
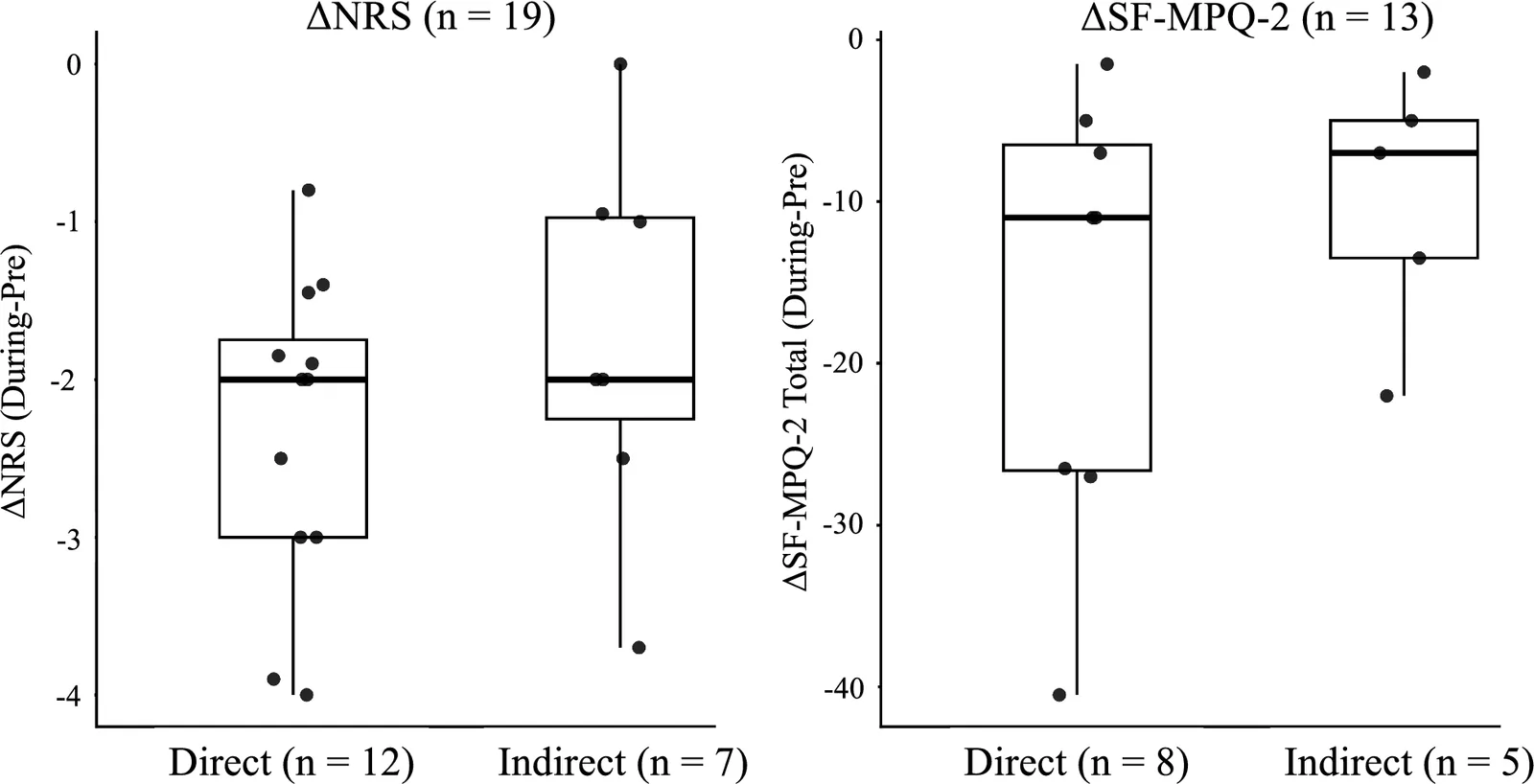
Aggregated change scores in dysesthesia intensity (NRS) and pain quality (SF-MPQ-2) during DM-TENS (During–Pre). Each point represents an aggregated unit of stimulation (subject × pain region × condition), showing change scores (During–Pre) for the Numerical Rating Scale (ΔNRS) (left) and the Short-Form McGill Pain Questionnaire–2 total score (ΔSF-MPQ-2) (right). Negative values denote symptom reduction during stimulation. For ΔNRS, the 19 aggregated units comprised 12 under the direct stimulation condition and 7 under the indirect stimulation condition; for ΔSF-MPQ-2, 13 aggregated units comprised 8 under the direct stimulation condition and 5 under the indirect stimulation condition. Boxplots indicate the median and interquartile range; whiskers extend to 1.5 × the interquartile range. Aggregated ΔNRS values were derived only from sessions with complete paired NRS data, whereas aggregated ΔSF-MPQ-2 values additionally included two indirect facial sessions with SF-MPQ-2 only and no concurrent NRS

Aggregated pre–during comparisons indicated a reduction in NRS scores during stimulation, with a median NRS change of − 2.0 points (IQR − 2.75 to − 1.43). The Wilcoxon signed-rank test demonstrated a statistically significant difference (V = 171, *p* = 0.00021), with a 95% confidence interval (95% CI) for the median difference of − 2.85 to − 1.85, corresponding to an effect size of r = 0.85. Comparison between direct and indirect stimulation using the Mann–Whitney U test did not detect a statistically significant difference in ΔNRS between conditions (n = 12 for direct stimulation, n = 7 for indirect stimulation, W = 31.5, *p* = 0.40) (Fig. 3). Because stimulation condition was structurally confounded with pain region, the condition-related null effects in the mixed-effects model and the null between-condition result in the aggregated analysis should not be interpreted as evidence of comparable effects between direct and indirect stimulation.

One case of acute Bell’s palsy, differing in temporal profile and pathophysiology, was included in the dataset. Exclusion of this case did not materially alter the results. The main effect of session in the linear mixed-effects model remained unchanged (estimate − 2.28, *p* < 0.001), and aggregated analysis showed a similar median reduction in NRS (− 2.0; V = 171, *p* = 0.00021 vs. V = 153, *p* = 0.00032).

### Responder analyses of NRS

Responder rates based on three exploratory responder thresholds are shown in Supplementary Table S2 (Additional File 1). At the session level (64 sessions), responder proportions were 84.4% for a ≥ 30% reduction in NRS, 67.2% for a ≥ 50% reduction, and 59.4% for an absolute reduction of ≥ 2 points. At the aggregated unit level (19 subject–pain region–condition units), the corresponding responder proportions were 89.5%, 73.7%, and 57.9%, respectively.

### Changes in pain quality (SF-MPQ-2) during DM-TENS

**Fig. 4:**
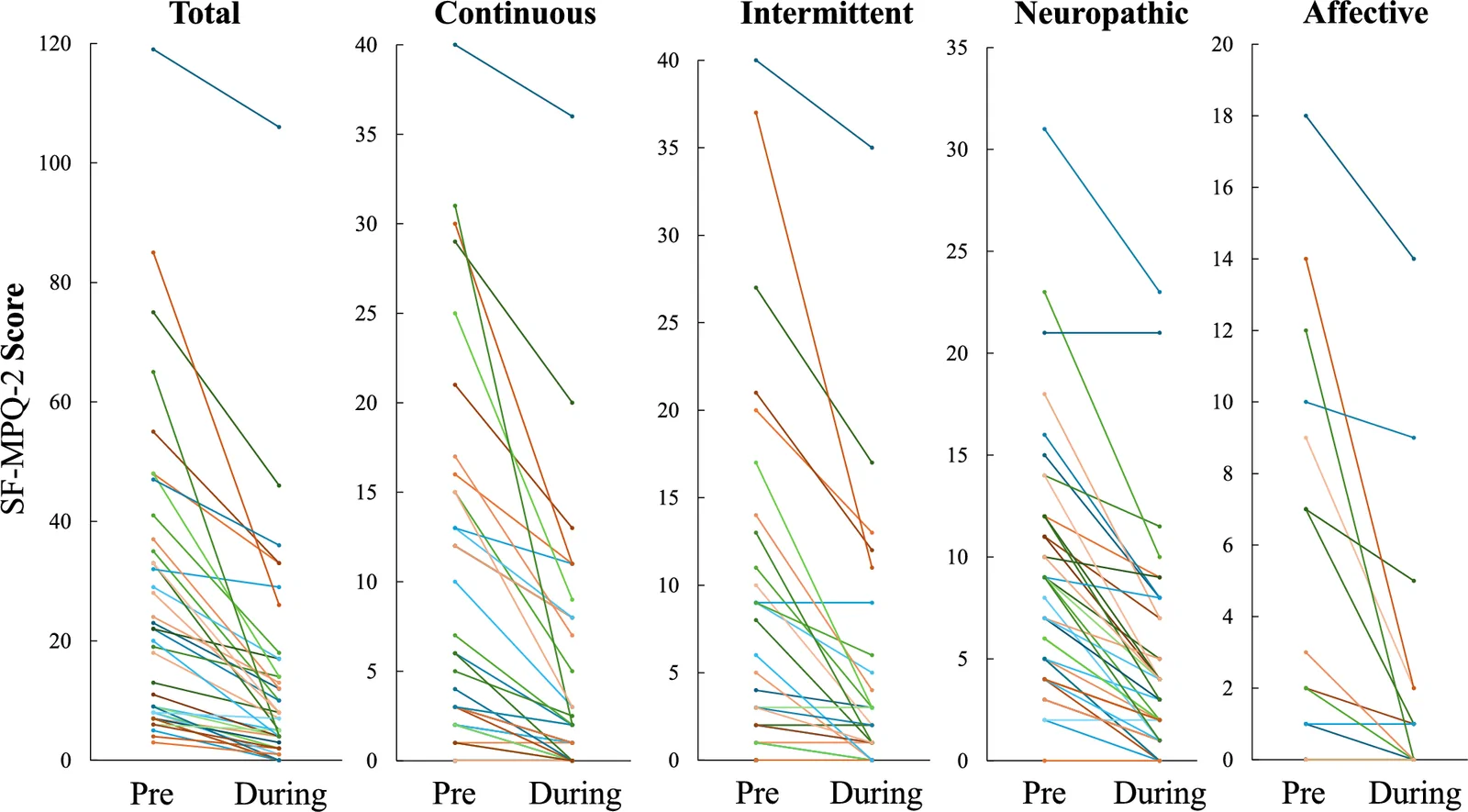
Session-level changes in pain quality domains assessed by the SF-MPQ-2 before (Pre) and during (During) DM-TENS. Paired lines represent individual stimulation sessions (total: 40 sessions), showing Short-Form McGill Pain Questionnaire–2 (SF-MPQ-2) scores immediately before (Pre) and during stimulation (During) across five domains: Total, Continuous, Intermittent, Neuropathic, and Affective. As the SF-MPQ-2 was originally developed for longer recall periods, its use for immediate within-session assessment in this study was exploratory

Session-level changes in pain-related sensory descriptors assessed using the SF-MPQ-2 are shown in Fig. 4.

### Session-level mixed-effects model

A linear mixed-effects model including all available session-level SF-MPQ-2 total scores showed a significant main effect of session, with lower scores during stimulation compared with pre-stimulation (estimate − 17.46, SE 5.67, t =  − 3.08, *p* = 0.002). Neither the main effect of stimulation condition (*p* = 0.12) nor the session × condition interaction (*p* = 0.35) reached statistical significance.

### Aggregated non-parametric sensitivity analyses

The aggregated ΔSF-MPQ-2 total demonstrated a median change of –11 points (IQR − 22 to − 5). The Wilcoxon signed-rank test showed a statistically significant within-unit change (V = 91, *p* = 0.0016), with a 95% CI for the median location shift of − 23.75 to − 5.50, corresponding to an effect size of r = 0.88. Analyses of individual SF-MPQ-2 subscales showed reductions in continuous, intermittent, and neuropathic pain descriptors. After adjustment for multiple comparisons using the Holm–Bonferroni method, these reductions remained statistically significant for the continuous (adjusted *p* = 0.011), intermittent (adjusted *p* = 0.018), and neuropathic (adjusted *p* = 0.010) subscales, whereas the affective descriptors did not reach statistical significance (adjusted *p* = 0.058). Comparisons between direct and indirect stimulation using the Mann–Whitney U test did not detect a statistically significant difference in ΔSF-MPQ-2 total score (*p* = 0.50) (Fig. 3). However, because stimulation condition was structurally confounded with pain region, the condition-related null effects in the mixed-effects model and the null between-condition result in the aggregated analysis should not be interpreted as evidence of comparable effects between stimulation conditions.

### Stimulation parameters across pain regions

Across pain regions, the median stimulation frequency, pulse width, and stimulation intensity were 150 Hz (IQR: 50–200 Hz), 60 μs (IQR: 50–70 μs), and 22 mA (IQR: 15–32 mA), respectively.

In exploratory session-level linear mixed-effects models, stimulation frequency did not show statistically significant regional variation (F(2, 64.39) = 1.52, *p* = 0.226), whereas pulse width and stimulation intensity differed across pain regions, with the magnitude of variation greater for stimulation intensity (pulse width: F(2, 55.45) = 5.71, *p* = 0.00557; intensity: F(2, 65.27) = 22.77, *p* = 3.14 × 10⁻^8^).

**Fig. 5:**
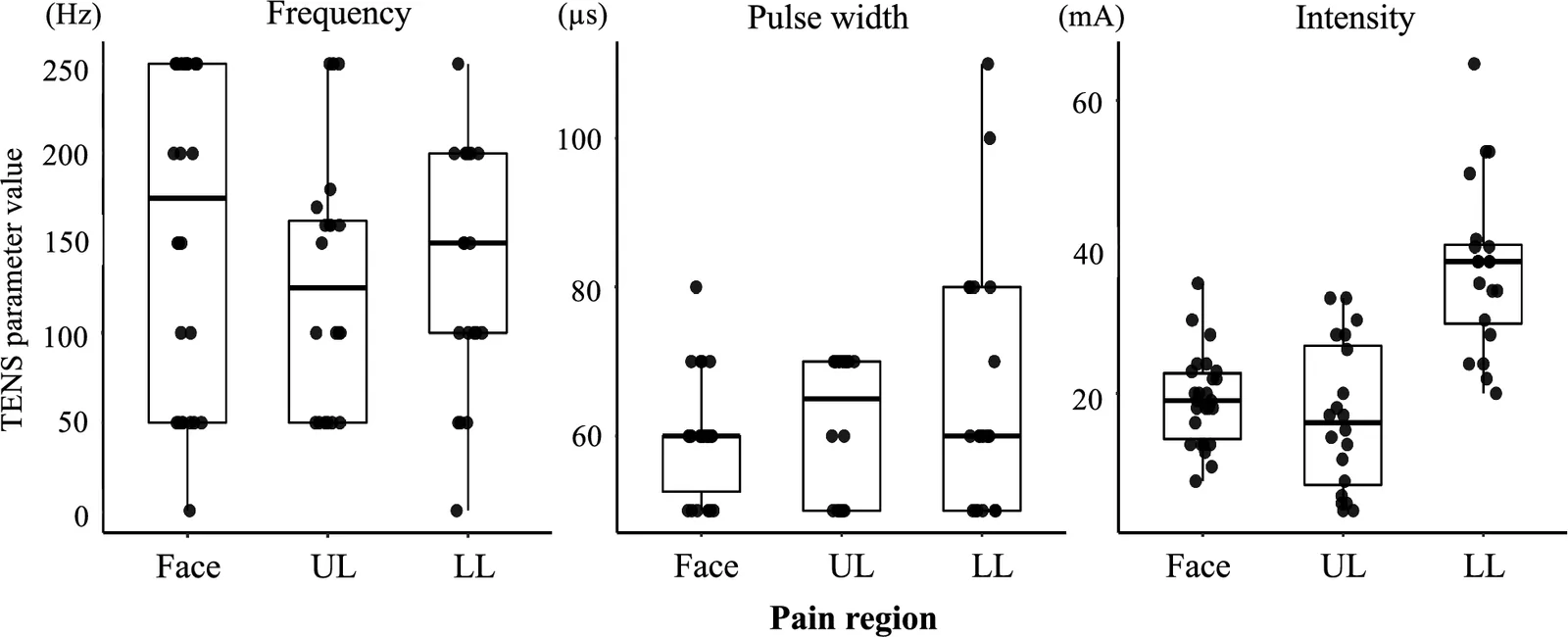
Distributions of DM-TENS stimulation parameters across pain regions. Boxplots and jittered points illustrate the distributions of stimulation frequency (Hz), pulse width (µs), and intensity (mA) used during DM-TENS sessions for different anatomical pain regions, namely the face (Face; 26 sessions), upper limb (UL; 20 sessions), and lower limb (LL; 20 sessions). Frequency and pulse width showed similar distributions across regions, whereas stimulation intensity values tended to be higher in the LL compared with those in the Face and UL regions. Sporadically higher pulse-width values were observed in the LL region

In exploratory sensitivity analyses using subject-by-region median values, stimulation frequency and pulse width did not differ significantly across regions (frequency: χ^2^ = 0.658, df = 2, *p* = 0.720; pulse width: χ^2^ = 0.718, df = 2, *p* = 0.699). In contrast, stimulation intensity differed across regions (χ^2^ = 10.597, df = 2, *p* = 0.005), with post hoc comparisons indicating higher values in the lower limb compared with the face and upper limb (Bonferroni-adjusted *p* = 0.018 and *p* = 0.035, respectively). The distributions of stimulation parameters used during DM-TENS sessions across different pain regions are shown in Fig. 5.

### Associations between stimulation parameters and symptom measures

Exploratory Spearman correlation analyses did not indicate clear associations between stimulation parameters (frequency, pulse width, and intensity) and baseline symptom severity, either for baseline NRS scores (|ρ|≤ 0.21, all *p* > 0.09) or for baseline SF-MPQ-2 scores (|ρ|≤ 0.19, all *p* > 0.23). Likewise, these analyses did not indicate clear associations between stimulation parameters and ΔNRS (|ρ|< 0.23, all *p* > 0.07) or ΔSF-MPQ-2 total scores (|ρ|< 0.15, all *p* > 0.36) (Supplementary Table S3, Additional File 1).

### Analyses by neuropathic pain type

In 64 stimulation sessions from 15 participants, exploratory analysis by neuropathic pain type did not identify a statistically significant difference in ΔNRS across pain types (Supplementary Fig. S3, Additional File 2). Session-level linear mixed-effects analysis showed no statistically significant pain-type-related difference (F(2, 33.38) = 0.73, *p* = 0.49). Subject-level aggregated median ΔNRS values also did not differ significantly across groups (Kruskal–Wallis χ^2^ = 1.59, df = 2, *p* = 0.45). Similar results were obtained after adjustment for stimulation condition and anatomical pain region (all *p* > 0.38).

### Consistency of stimulation parameters

The exploratory consistency analyses included 62 sessions from 11 patients, forming 15 subject–region clusters with repeated measurements. Median absolute changes across sessions were 0 Hz for stimulation frequency, 0 µs for pulse width, and 2 mA for stimulation intensity. ICC(1,1) values (95% CI) were 0.818 (0.717–0.879) for stimulation frequency, 0.789 (0.676–0.856) for pulse width, and 0.850 (0.762–0.900) for stimulation intensity (Supplementary Table S4, Additional File 1).

## Discussion

This retrospective observational case series evaluated immediate within-session changes during dysesthesia-matched transcutaneous electrical nerve stimulation (DM-TENS) in patients referred from a pain clinic, most of whom had chronic or treatment-refractory neuropathic pain. The main finding was that lower within-session NRS scores were observed, accompanied by exploratory changes in pain quality as reflected in the SF-MPQ-2 total score and several subscale domains. These changes were observed across a clinically heterogeneous cohort spanning trigeminal-, spinal-, and peripheral-origin neuropathic pain and different stimulation configurations. DM-TENS was well tolerated, with no session discontinuations due to discomfort and no serious adverse effects observed, supporting its feasibility in routine clinical practice.

Given that this case series lacked a sham or control condition, these within-session changes cannot establish a causal treatment effect, and placebo effects, expectancy, or other non-specific contextual influences cannot be excluded. In addition, although lower symptom scores were observed across neuropathic pain types and stimulation configurations, these patterns should be interpreted descriptively and not as evidence of comparable effects between direct and indirect stimulation, because stimulation condition was completely confounded with pain region. The modest and heterogeneous cohort also limits generalizability to broader neuropathic pain populations.

Exploratory responder analyses were performed to facilitate clinical interpretation of immediate within-session changes in dysesthesia intensity. Using IMMPACT thresholds as primary responder definitions, a proportion of sessions met criteria for ≥ 30% and ≥ 50% reductions in NRS (Supplementary Table S2, Additional File 1). In sensitivity analyses based on the anchor-based ≥ 2-point threshold, approximately 60% of sessions also met this criterion. However, as these responder definitions were originally developed for longer-term treatment outcomes [17, 18], these responder analyses should be considered exploratory and interpreted with caution.

Collectively, these findings suggest that DM-TENS is feasible and may be associated with short-term modulation of dysesthetic symptoms in routine care settings, warranting further evaluation in controlled prospective studies.

### Clinical implications of immediate symptom changes

The lower within-session symptom scores observed in this study suggest that DM-TENS may be feasible as a non-invasive approach for patients with refractory neuropathic pain.

In the present case series, some patients reported transient, subjective improvements in motor function concurrent with reductions in abnormal sensations and pain during stimulation. For example, patients described transient changes in gait, manual dexterity, or orofacial control. Although anecdotal and hypothesis-generating, these observations suggest a possible link between short-term sensory changes during DM-TENS and transient changes in sensorimotor integration. This interpretation is consistent with a recent case report describing immediate improvements in quantitative measures of hand dexterity and force control following DM-TENS synchronized with neuropathic pain characteristics [19].

The responsiveness to rehabilitation interventions may depend on the neurophysiological and perceptual state at the time the intervention is initiated, a concept discussed in the context of neuroplasticity [20]. Preparatory modulation–often referred to as sensory or neural priming [21]– involves transient changes in sensory processing that may increase responsiveness to subsequent motor training or functional activities. Short-term sensory changes may reduce interference from ongoing pain and abnormal sensory input and may thereby facilitate movement practice and rehabilitation engagement. Rather than demonstrating a direct rehabilitation effect, and given that functional outcomes were not directly assessed, the present findings raise the testable hypothesis that transient modulation of dysesthesia by DM-TENS may create a temporary sensory state relevant to subsequent rehabilitation and motor recovery, consistent with concepts of sensory priming and state-dependent neuroplasticity [21, 22].

### Pain quality modulation beyond intensity

Alongside the lower NRS scores, the exploratory SF-MPQ-2 assessment showed decreases in the total score and several subscales, including continuous, intermittent, and neuropathic pain descriptors, whereas changes in the affective subscale were less pronounced. One possible explanation is that affective descriptors may be less responsive to brief sensory modulation and may require sustained improvement or repeated sessions to change.

In addition, the therapeutic concept of DM-TENS is not to adjust electrical stimulation solely according to pain intensity, but rather to incorporate the qualitative features of dysesthesia into parameter adjustment. Moreover, this view aligns with contemporary models that conceptualize neuropathic pain as a disorder of altered sensory processing rather than a simple amplification of nociceptive input [23, 24]; however, this interpretation remains speculative.

In conventional TENS trials, stimulation parameters such as frequency and pulse width are commonly prespecified, while adjustment during each session typically focuses on stimulation intensity and tolerability within predefined ranges [25–31]. In contrast, DM-TENS uses perceptual matching, in which patient feedback guides iterative adjustment of stimulation parameters and electrode placement so that the evoked sensation approximates the patient’s dysesthetic experience. This approach may provide a framework for tailoring cutaneous electrical stimulation to symptom quality and warrants further comparative evaluation.

### Mechanistic considerations and perceptually guided neuromodulation

The within-session changes observed during DM-TENS may be compatible with several mechanisms proposed for TENS analgesia [32], rather than a single causal pathway. Proposed mechanisms include spinal segmental inhibition (“gating”), modulation of peripheral input, endogenous pain-modulating systems, including descending pain inhibitory pathways such as the PAG–RVM system [32–40].

A recent study has also suggested a possible role for a dorsal column–thalamocortical circuit involving Aβ low-threshold mechanoreceptors (Aβ-LTMRs) in TENS-related analgesia [41].

The relative contribution of these mechanisms may vary depending on stimulation mode and intensity [36]. However, the present retrospective clinical data cannot determine which mechanisms contributed to the observed symptom changes.

DM-TENS differs conceptually from fixed-parameter approaches because stimulation is adjusted in real time according to the patient’s dysesthetic perception. Given that dysesthesia and neuropathic pain can be viewed as altered sensory processing rather than passive nociceptive transmission [23, 24], the observed changes in symptom intensity and pain-quality descriptors may be compatible with short-term modulation of sensory processing. This interpretation should be regarded as hypothesis-generating and requires confirmation using objective neurophysiological measures.

### Indirect stimulation: remote application of DM-TENS

In patients with trigeminal neuropathic symptoms, indirect stimulation delivered to a remote site was associated with lower within-session symptom scores.

This indirect approach originated from an incidental observation in an index patient who received direct DM-TENS for postoperative L5-related dysesthesia. During lower-limb stimulation, the patient reported concurrent changes in chronic facial symptoms within the V2–V3 distribution, despite the absence of facial stimulation. In subsequent visits, similar patient-reported changes in facial symptoms were observed during remote stimulation to the palm or lower leg, and palmar stimulation was adopted because it was clinically more accessible and required lower stimulation intensity than lower-limb stimulation.

Based on this observation, palmar stimulation was subsequently applied in later patients with trigeminal neuropathic pain. During parameter tuning, some patients reported a sensation of stimulation “spreading” to the facial region or subjective loosening of the symptomatic area while stimulation was delivered to the palm and adjusted based on facial dysesthesia. These subjective cues served as pragmatic indicators during adjustment. In contrast, one patient with V1-dominant post-herpetic trigeminal neuropathy showed a limited response, suggesting that responsiveness to stimulation may vary depending on symptom phenotype, neural substrates, or the feasibility of achieving perceptual matching.

The observations with indirect stimulation raise mechanistic questions regarding how remote sensory input might be associated with changes in trigeminal symptoms. Because stimulation was delivered outside the symptomatic facial region, broader sensory integration or central pain-modulating mechanisms may be relevant. A meta-analysis supports the clinical plausibility of TENS for trigeminal pain when applied directly to the face [42]. Remote analgesic effects of conventional TENS have also been reported in other pain conditions, suggesting that mechanisms beyond strictly segmental modulation may be relevant [43]. However, the present findings do not establish comparable effects between direct and indirect stimulation, and the indirect stimulation observations should be regarded as hypothesis-generating.

Although the palm is anatomically distant from the trigeminal territory, it occupies a relatively large somatosensory cortical representation [44, 45]; moreover, neuroimaging and neurophysiological studies have described interactions between hand and face representations in pain states [46–48]. These findings provide a possible framework for future mechanistic studies, but the present clinical data cannot determine whether such cortical interactions contributed to the observed symptom changes.

Related work in brachial plexus avulsion has reported that TENS applied to referred sensation areas, such as ipsilateral cheek or shoulder, can evoke phantom touch sensations in the deafferented limb and may be associated with short-term analgesia [49]. This finding provides another example in which remote sensory stimulation may interact with altered sensory representations, although its relevance to trigeminal neuropathic symptoms during DM-TENS remains uncertain.

These findings should be considered preliminary and should not be generalized beyond the limited face–palm configuration examined here, because indirect stimulation was completely confounded with pain region. The observations suggest that remote stimulation may warrant further investigation in selected trigeminal neuropathic presentations when perceptual matching can be achieved.

### Parameter tuning in DM-TENS

In this study, stimulation frequency, pulse width, and intensity varied across individuals and pain regions, and analyses did not identify consistent associations between any single parameter and the symptom change. These findings suggest that the observed within-session changes were unlikely to be explained by an isolated parameter setting alone. This interpretation is broadly consistent with other neuromodulation approaches, such as scrambler therapy, in which diverse stimulation patterns—rather than fixed settings—are thought to contribute to analgesic efficacy across patients [50]. In DM-TENS, parameter adjustment may be better conceptualized as a perceptually guided process in which stimulation settings are iteratively selected based on the patient’s individual sensory experience.

Higher stimulation intensities observed in the lower limb should not be interpreted simply as tolerance-driven maximal output. They may reflect anatomical and sensory factors, such as tissue thickness, neural density, and regional sensory thresholds, and the perceived strength, spatial extent, and qualitative features of dysesthesia. Thus, stimulation intensity might be better understood as a parameter shaped by perceptual feedback rather than a predetermined or region-restricted target.

Although stimulation parameters were repeatedly adjusted based on subjective patient feedback, consistency analyses suggested that the selected settings showed relative within-patient consistency across repeated sessions within the same pain region. Intraclass correlation coefficients ranged from 0.78 to 0.85, indicating good to excellent consistency. Meanwhile, optimal settings were not always identified immediately, and sometimes shifted as symptoms evolved. Taken together, these findings suggest that although DM-TENS settings are inherently dynamic and individualized, they may show relative consistency within the same patient over time, supporting the practical feasibility of parameter adjustment in clinical settings, without implying standardization across patients.

Future work should incorporate objective physiological measures—such as somatosensory evoked potentials or indices of cortical excitability—to examine whether neurophysiological changes accompany within-session symptom changes and to clarify the mechanisms underlying perceptually guided parameter adjustment.

### Limitations

Several limitations should be acknowledged.

First, this study was designed as a retrospective observational case series without a sham or control condition; therefore, placebo effects, expectancy, regression to the mean, and other non-specific contextual influences cannot be excluded.

Second, approximately half of the sessions relied on converted VAS values rather than directly measured NRS scores. Although linear rescaling is an accepted harmonization approach, the relatively high proportion of converted data may have influenced the results and should be considered when interpreting the findings.

Third, the sample size was modest and the cohort clinically heterogeneous, including trigeminal, spinal, and peripheral neuropathic pain. Although this heterogeneity reflects real-world clinical practice, it limits generalizability to broader neuropathic pain populations, as well as inference regarding diagnosis-specific responses, robust subgroup analyses, and reliable predictors of response.

Fourth, the SF-MPQ-2 was originally developed to assess pain symptoms over the preceding week [13]. In the present study, it was administered immediately before and during stimulation to capture acute qualitative changes. Although this approach was intended to capture acute changes in pain quality, the validity and responsiveness of SF-MPQ-2 for ultra-short–term assessment have not been formally established. Future prospective studies should therefore incorporate assessment tools specifically validated for momentary or near-real-time evaluation of sensory descriptors.

Fifth, the present analyses were limited to within-session changes. Although patient-reported persistence of symptom change was summarized descriptively, the durability of symptom changes, potential cumulative benefits with repeated DM-TENS sessions, and longer-term functional outcomes—including activity levels, motor performance, and quality of life—were not assessed. Prospective studies with standardized follow-up are required to determine whether the immediate changes observed here are associated with sustained clinical benefit.

Sixth, although lower within-session symptom scores were observed during indirect stimulation, inter-individual variability was observed, and the generalizability of indirect DM-TENS across all trigeminal or orofacial presentations cannot be assumed. In addition, palmar stimulation was applied to either the ipsilateral or contralateral hand relative to the symptomatic side, and the potential influence of stimulation laterality—including ipsilateral, contralateral, or bilateral palmar stimulation—was not well examined. Differences in clinical responses related to stimulation laterality therefore cannot be excluded and warrant further investigation.

Finally, although DM-TENS was generally well tolerated, transient symptom exacerbation was observed in one case after an initial decrease in symptoms. Such responses may reflect inter-individual differences in sensory responsiveness or altered sensory processing, and increased activity after an initial decrease in symptoms may also have contributed. This underscores the need for careful monitoring and guidance regarding activity levels after DM-TENS.

Future investigations should employ prospective controlled designs—including randomized, crossover, or N-of-1 approaches—and integrate mechanistic measurements such as quantitative sensory testing, neurophysiological markers, or neuroimaging. Such studies will be necessary to examine the potential perceptual and neurophysiological mechanisms, explore predictors of response, and inform clinical implementation of DM-TENS.

## Conclusion

This retrospective, observational case series showed lower within-session symptom scores during dysesthesia-matched transcutaneous electrical nerve stimulation (DM-TENS) across heterogeneous neuropathic pain conditions, including trigeminal, spinal, and peripheral neuropathic pain presentations. DM-TENS was well tolerated and feasible in routine clinical practice.

The observations regarding indirect stimulation, particularly for trigeminal neuropathic presentations, should be considered preliminary because stimulation condition was confounded with pain region and patient subgroup. These preliminary findings warrant further evaluation in prospective controlled studies using unconfounded designs. Future studies incorporating mechanistic outcomes, such as neurophysiological measures, as well as functional outcomes, are needed to better understand the clinical significance and underlying mechanisms of DM-TENS.

## Supplementary Information

Additional file 1. This file contains detailed tables of patient characteristics and results of additional analyses that are not included in the main manuscript.

Additional file 2. This file contains supplementary figures, including a flowchart of the DM-TENS parameter adjustment procedure, a schematic overview of the analyzable datasets and aggregation structure, and the distribution of ΔNRS across neuropathic pain types.

## Data Availability

The datasets are available from the corresponding author on reasonable request.

## Abbreviations

DM-TENS: Dysesthesia-matched transcutaneous electrical nerve stimulation
Direct: Direct stimulation
Indirect: Indirect stimulation
ICC: Intraclass correlation coefficient
LMM: Linear mixed-effects model
NRS: Numerical rating scale
SF-MPQ-2: Short-form McGill pain questionnaire-2
VAS: Visual analogue scale
V1/V2/V3: Ophthalmic/Maxillary/Mandibular divisions of trigeminal nerve
UL: Upper limb
LL: Lower limb
