# Supplementary material for: Immediate changes during dysesthesia-matched transcutaneous electrical nerve stimulation in refractory neuropathic pain: a retrospective observational case series
Source: J Neuroeng Rehabil. 2026 Jul 3;23:281. doi: 10.1186/s12984-026-02084-6 (PMC13602798; doi:10.1186/s12984-026-02084-6)
Supplement: Supplementary file 1 [file 12984_2026_2084_MOESM2_ESM.pdf]

Supplementary Figures

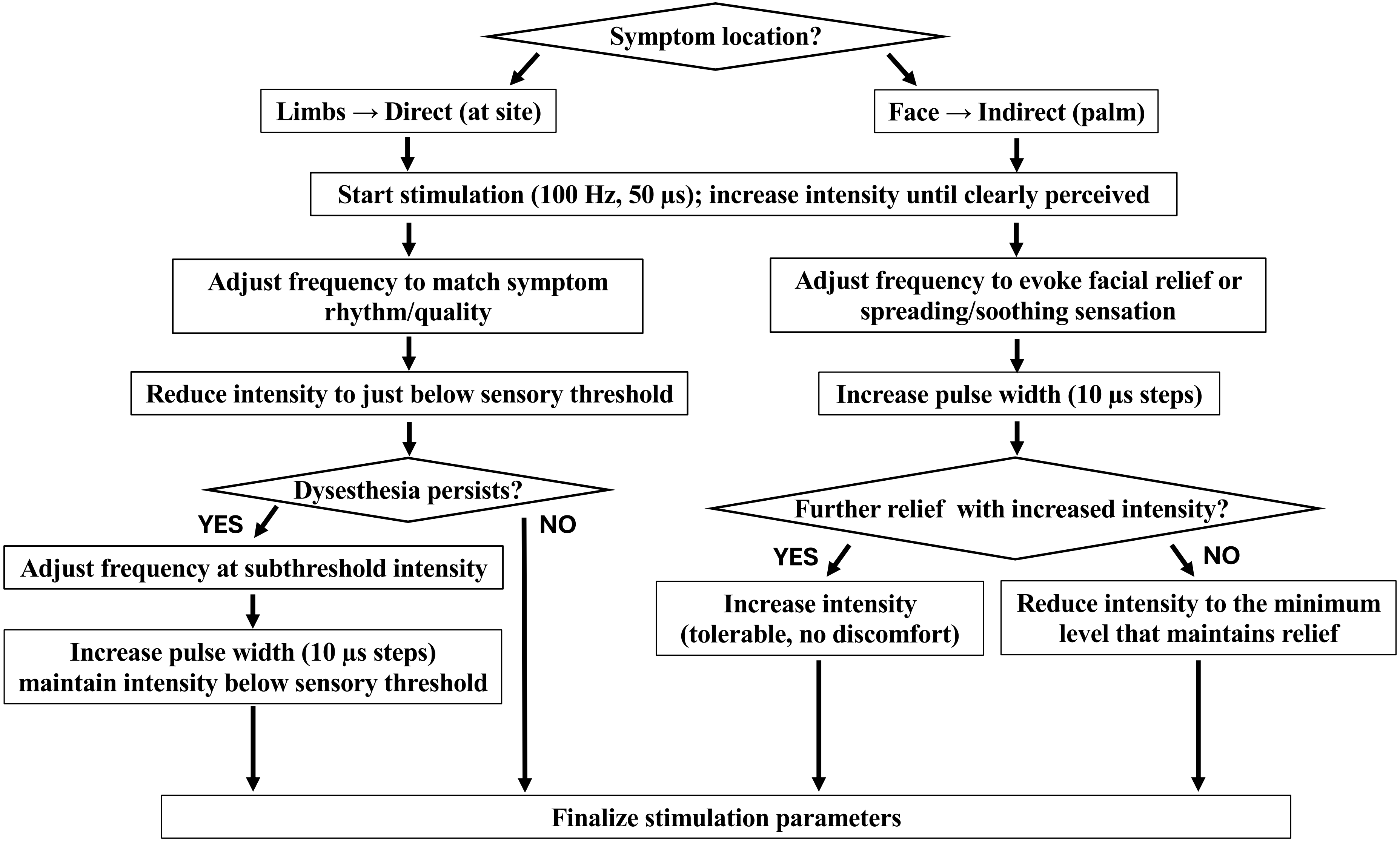

Fig. S1. Flowchart of the DM-TENS parameter adjustment procedure.

This figure summarizes the stepwise procedure used to optimize stimulation parameters for

(1) direct stimulation of symptomatic limb regions and

(2) indirect palm stimulation for facial symptoms within the trigeminal nerve distribution.

In both approaches, stimulation was initiated at 100 Hz and 50 µs, and frequency was adjusted before pulse width. Parameter optimization was guided by the patient's real-time verbal feedback regarding symptom relief and tolerability.

Direct stimulation

Electrodes were placed over the symptomatic region. Stimulation was initiated at 100 Hz and 50 µs, and current intensity was gradually increased until the patient clearly perceived the electrical sensation. Frequency was then adjusted to match the perceived rhythm or qualitative features of the dysesthesia. Once the dysesthesia diminished or disappeared, current intensity was progressively reduced to a level slightly below the sensory threshold. If dysesthesia remained, frequency was further adjusted at subthreshold intensity to identify the optimal setting for symptom relief. When further residual symptoms remained, pulse width was increased in 10-µs steps to further optimize symptom alleviation.

Indirect stimulation for facial dysesthesia / pain

Electrodes were placed on the palm of the ipsilateral or contralateral hand. As in direct stimulation, stimulation was initiated at 100 Hz and 50 µs, and current intensity was increased until a clear sensation was perceived in the palm. Frequency was then adjusted to identify settings associated with a perceived sense of relief or a spreading/soothing sensation in the facial region. When such facial sensations or symptom changes were not sufficiently elicited, current intensity at the palm was increased within tolerable limits. Pulse width was subsequently adjusted incrementally to further optimize symptom alleviation. Once maximal symptom alleviation was achieved, current intensity was gradually reduced to the lowest level at which this alleviation was maintained. In this

indirect approach, stimulation intensity was adjusted within tolerable limits and could be maintained above the sensory threshold when this was associated with greater facial symptom relief.

Notes

- “Intensity” refers to current intensity, (mA).
- At each adjustment step, parameter values were retained if they provided greater symptom relief. If no further benefit was observed, the previous effective setting was maintained.
- When changes in frequency or pulse width altered the perceived stimulation intensity, current intensity was adjusted as needed.
- Frequency was adjusted stepwise, using initial coarse increments of approximately ±50 Hz followed by finer tuning in 10–20 Hz increments, with a minimum step size of 10 Hz.
- Once optimal parameters were identified within a session, stimulation was continued without further adjustment.
- In patients with multiple sessions, stimulation parameters were re-optimized at each session rather than fixed across sessions.

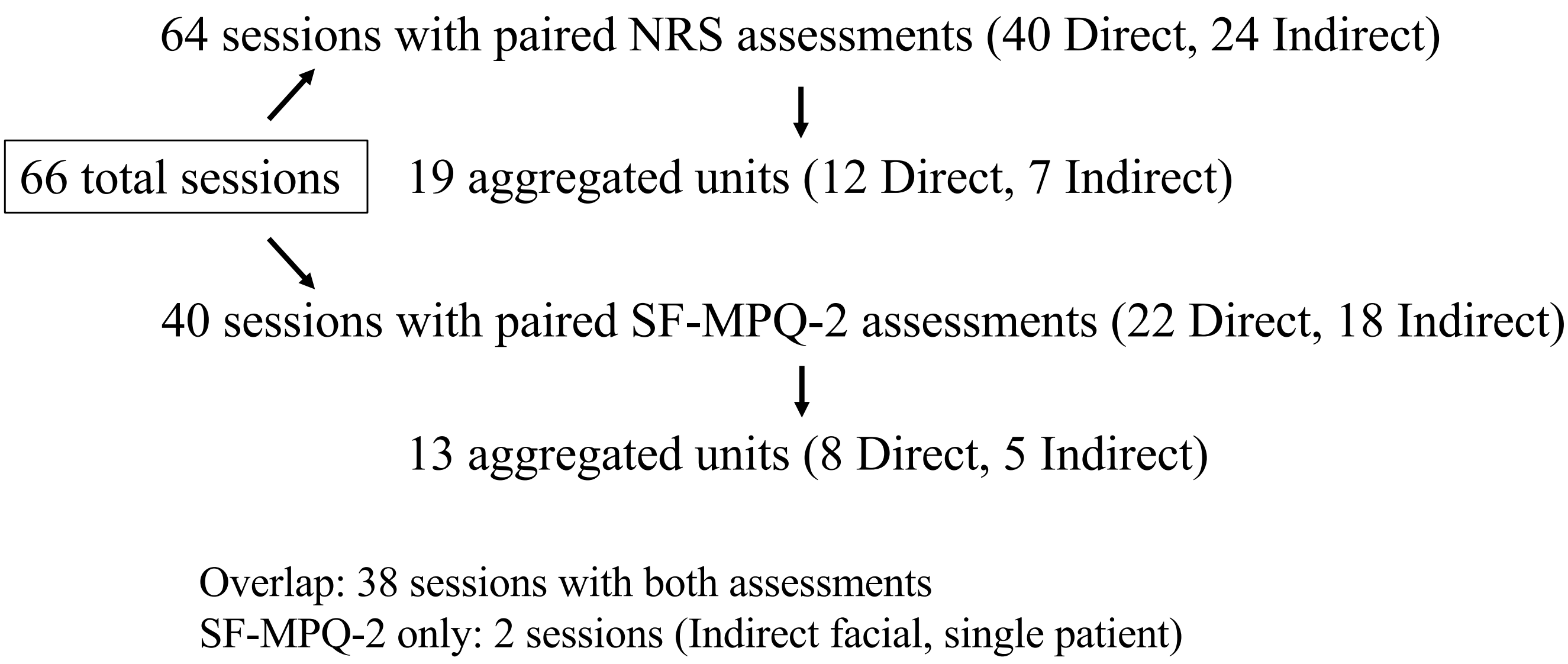

Fig. S2. Schematic overview of analyzable datasets and aggregation structure.

A total of 66 DM-TENS sessions were included. Of these, 64 sessions had paired pre–during NRS assessments, 40 had paired pre–during SF-MPQ-2 assessments, and 38 had both assessments available. Aggregation by subject × pain region × stimulation condition yielded 19 units for ΔNRS analyses, comprising 12 Direct and 7 Indirect stimulation units, and 13 units for ΔSF-MPQ-2 analyses, comprising 8 Direct and 5 Indirect stimulation units. Two sessions included SF-MPQ-2 assessments only, without concurrent NRS assessments; both were Indirect facial sessions from a single patient.

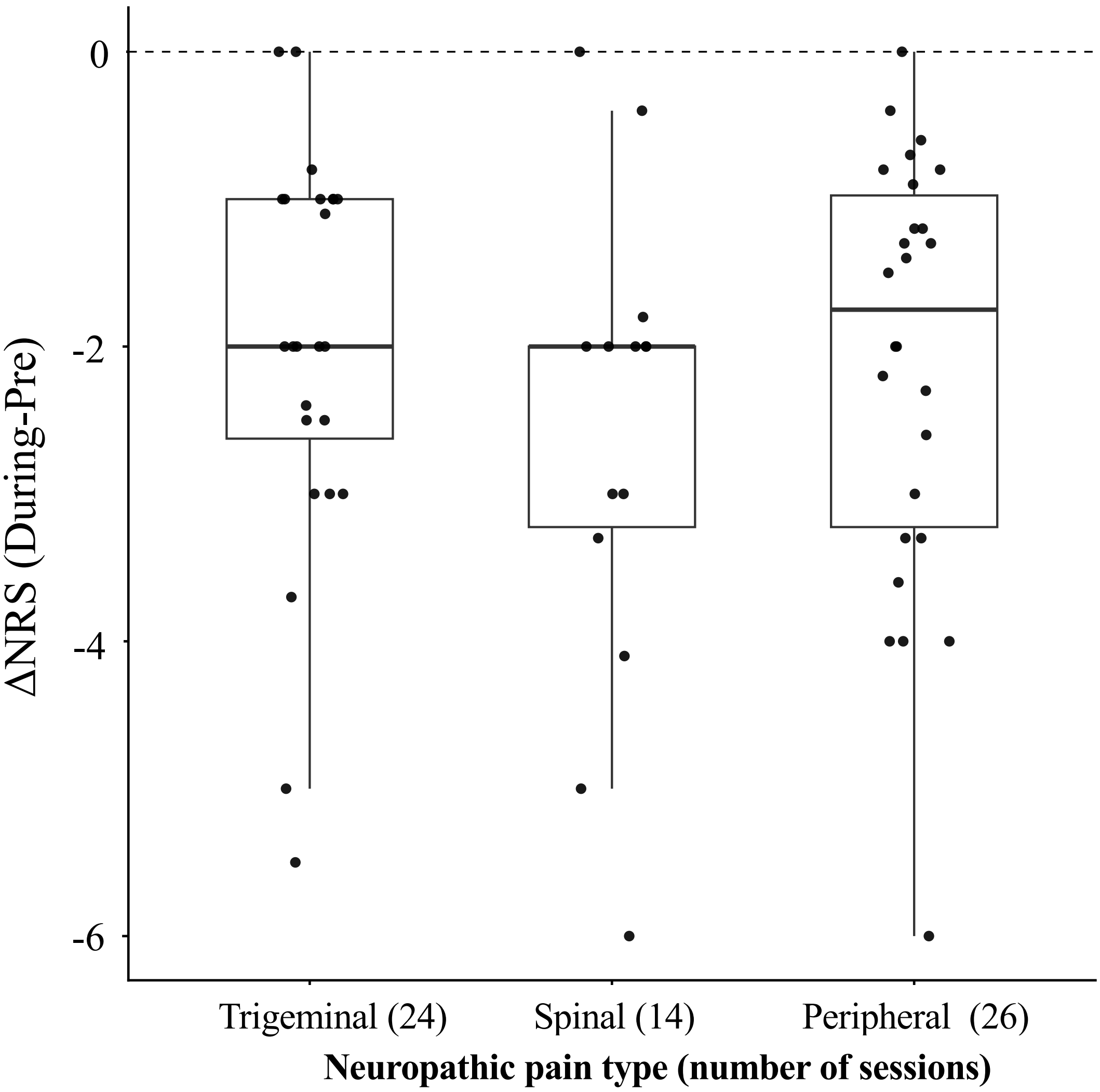

Fig. S3. Distribution of changes in Numerical Rating Scale (ΔNRS; During − Pre) across neuropathic pain types.

Each point represents an individual stimulation session. Boxplots indicate the median and interquartile range; whiskers extend to 1.5 × the interquartile range, with individual points representing all sessions. Session counts are shown in parentheses for each pain type. No clear differences in ΔNRS were observed among trigeminal, spinal, and peripheral neuropathic pain types, consistent with session-level linear mixed-effects models and subject-level aggregated analyses.
