# Supplementary material for: Immediate changes during dysesthesia-matched transcutaneous electrical nerve stimulation in refractory neuropathic pain: a retrospective observational case series
Source: J Neuroeng Rehabil. 2026 Jul 3;23:281. doi: 10.1186/s12984-026-02084-6 (PMC13602798; doi:10.1186/s12984-026-02084-6)
Supplement: Supplementary file 2 [file 12984_2026_2084_MOESM1_ESM.docx]

**Supplementary Tables**

| **Table S1.** Patient, pain-region, and stimulation characteristics | | | | |  |  | |  |  |  |  |  |  | |  |  |
| --- | --- | --- | --- | --- | --- | --- | --- | --- | --- | --- | --- | --- | --- | --- | --- | --- |
|  |  |  |  |  | | |  | | **Number of sessions** | |  | **Parameter range** | | | |  |
| **Subject** | **Sex/Age** | **Diagnosis** | **Pain regions** | **Stim site** | | | **Symptom duration  (months)** | | **NRS** | **SF-MPQ**-**2** |  | **Frequency  (Hz)** | | **Pulse width  (µs)** | **Intensity  (mA)** | **Maximum persistence**  **of symptom change**  **(patient-reported, retrospective)** |
| 1 | F/62 | Lumbar disc herniation after surgery | L leg | L leg | | | 24 | | 4 | 1 |  | 1–50 | 50 | | 20–24 | ≤10 min |
|  |  | Trigeminal neuropathy | R V3 area | R palm | | | 156 | | 5 | 3 |  | 1–150 | 50 | | 10–24 | ≤10 min |
| 2 | M/76 | Chemotherapy-induced peripheral neuropathy | Bil feet | Bil feet | | | 98 | | 3 | 0 |  | 200–250 | 60–70 | | 34–41 | 1-7 days |
|  |  |  | Bil hand | Bil hand | | | 98 | | 2 | 0 |  | 250 | 60 | | 18-28 | 1-7 days |
| 3 | F/23 | Vaccine associated neuropathy | R arm | R arm | | | 2 | | 6 | 6 |  | 160–250 | 70 | | 4–8 | ≥1 week |
|  |  |  | Bil V2 area | R palm | | | 2 | | 2 | 2 |  | 50 | 50 | | 8–12 | ≥1 month |
| 4 | F/58 | Cervicothoracic disc herniation after surgery | R leg | R leg | | | 36 | | 2 | 2 |  | 150 | 50 | | 53 | ≤10 min |
|  |  |  | R hand | R hand | | | 36 | | 2 | 0 |  | 100 | 50 | | 17 | No persistence beyond stimulation |
| 5 | F/71 | Lumbar canal stenosis after surgery | R leg | R leg | | | 84 | | 1 | 0 |  | 150 | 100 | | 50 | No persistence beyond stimulation |
| 6 | F/26 | Trigeminal neuropathy after dental procedure | L V3 area | R palm | | | 12 | | 7 | 5 |  | 50–200 | 60–70 | | 16–22 | 1-7 days |
| 7 | M/72 | Trigeminal neuropathy after dental procedure | L V2, V3 area | L palm | | | 24 | | 3 | 1 |  | 250 | 60 | | 23–35 | ≥1 month |
| 8 | F/27 | Trigeminal neuropathy after dental procedure | Bil V3 area | L palm | | | 1 | | 5 | 7 |  | 250 | 60 | | 13–20 | 1-12h |
| 9 | F/78 | Drug-induced peripheral neuropathy | Bil feet | Bil feet | | | 72 | | 5 | 3 |  | 100 | 60 | | 30–38 | 1-12h |
| 10 | F/67 | Lumbar disc herniation after surgery | L leg | L leg | | | 28 | | 4 | 3 |  | 200 | 80 | | 28–40 | 1-12h |
| 11 | M/13 | Brachial plexus injury | R palm | R palm | | | 8 | | 3 | 2 |  | 50–100 | 50 | | 20–33 | ≥ 1week |
| 12 | M/65 | Compartment syndrome, causalgia | R forearm | R forearm | | | 7 | | 7 | 4 |  | 50–170 | 50–70 | | 11–33 | 1-12h |
| 13 | M/69 | Lumbar canal stenosis after surgery | Bil legs | Bil legs | | | 84 | | 1 | 1 |  | 200 | 110 | | 65 | ≤10 min |
| 14 | M/62 | Bell’s palsy with facial dysesthesia | R V2 area | R palm | | | 0 | | 1 | 0 |  | 200 | 70 | | 23 | ≥ 1month |
| 15 | M/51 | Herpetic neuralgia | L V1 area | L palm | | | 9 | | 1 | 0 |  | 200 | 80 | | 28 | No observable change |

In the first four rows, two distinct pain regions from the same patient are shown as separate entries. Pain region indicates the symptomatic area, whereas stimulation site denotes the anatomical location where DM-TENS was applied. Ranges of stimulation parameters reflect adjustments made across repeated sessions based on perceptual matching. Maximum persistence of symptom change represents the longest duration of patient-reported persistence of symptom change observed after stimulation, typically obtained retrospectively at subsequent visits. These observations were recorded descriptively without standardized follow-up assessment and were not used for inferential analyses.
**Abbreviations:** Bil, bilateral; L, left; R, right; Hz, hertz; µs, microseconds; mA, milliamperes.

| **Table S2.** Responder rates for immediate within-session changes in NRS | | | |
| --- | --- | --- | --- |
|  | **≥ 30% reduction** | **≥ 50% reduction** | **≥ 2-point reduction** |
| Session-level (64 sessions) (%) | 84.4 | 67.2 | 59.4 |
| Aggregated units (19 units) (%) | 89.5 | 73.7 | 57.9 |

Responder rates are shown for session-level data (64 sessions) and subject–pain region–condition aggregated units (19 units), in which repeated sessions were summarized using median values. Responders were defined as a ≥30% reduction in NRS (moderately important improvement) or a ≥50% reduction (substantial improvement), consistent with IMMPACT benchmarks [17], and as an absolute reduction of ≥2 points based on anchor-based thresholds [18]. Percentage reduction was calculated as (Pre − During) / Pre × 100. Responder analyses were exploratory and intended to facilitate clinical interpretation of immediate within-session changes in NRS.

| **Table S3. Correlations between baseline symptom severity or symptom changes and DM-TENS stimulation parameters** | | | | | | | | |
| --- | --- | --- | --- | --- | --- | --- | --- | --- |
|  | **Frequency (Hz)** | |  | **Pulse width (µs)** | |  | **Intensity (mA)** | |
| Scales | ρ | p |  | ρ | p |  | ρ | p |
| Pre NRS | 0.05 | 0.696 |  | 0.08 | 0.547 |  | 0.21 | 0.099 |
| Pre SF-MPQ-2 total | -0.05 | 0.759 |  | -0.09 | 0.586 |  | -0.19 | 0.237 |
| ΔNRS | -0.08 | 0.519 |  | 0.03 | 0.837 |  | -0.23 | 0.073 |
| ΔSF-MPQ-2 total | 0.03 | 0.879 |  | 0.14 | 0.389 |  | 0.15 | 0.369 |

Correlation analyses were performed using all available sessions for each outcome measure (NRS: 64 sessions; SF-MPQ-2: 40 sessions). Values represent Spearman’s rank correlation coefficients (ρ) with corresponding p values. Baseline measures include pre-stimulation Numerical Rating Scale (Pre NRS) scores and pre-stimulation total Short-Form McGill Pain Questionnaire-2 (Pre total SF-MPQ-2) scores. Δ values represent changes calculated as During − Pre; negative values indicate symptom reduction. No clear correlations were observed between symptom measures and stimulation parameters.

| **Table S4. Consistency of DM-TENS stimulation parameters across repeated sessions** | | | | |  |
| --- | --- | --- | --- | --- | --- |
| **Parameter** | **Sessions (n)** | **Clusters (n)** | **Median absolute change** | **ICC(1,1)** | **95% CI** |
| Frequency (Hz) | 62 | 15 | 0 | 0.818 | 0.717–0.879 |
| Pulse width (µs) | 62 | 15 | 0 | 0.789 | 0.676–0.856 |
| Intensity (mA) | 62 | 15 | 2 | 0.85 | 0.762–0.900 |

Intraclass correlation coefficients (ICC) were estimated using random-intercept models; clusters consisted of repeated sessions per subject–region unit with unequal numbers of sessions.
